# Role of glial 14-3-3 gamma protein in autoimmune demyelination

**DOI:** 10.1186/s12974-015-0381-x

**Published:** 2015-10-06

**Authors:** De-Hyung Lee, Petra Steinacker, Silvia Seubert, Tanja Turnescu, Arthur Melms, Arndt Manzel, Markus Otto, Ralf A. Linker

**Affiliations:** Department of Neurology, Friedrich-Alexander-University Erlangen-Nuremberg, Schwabachanlage 6, D-91054 Erlangen, Germany; Department of Neurology, Universitäts- und Rehabilitationskliniken Ulm (RKU), Oberer Eselsberg 45, D-89081 Ulm, Germany; Neurological Rehabilitation, Department of Neurology, Friedrich-Alexander-University Erlangen-Nuremberg, Schwabachanlage 6, D-91054 Erlangen, Germany

**Keywords:** Multiple sclerosis, Knockout mouse, Apoptosis, Oligodendrocyte, Astrocyte, MOG-EAE

## Abstract

**Background:**

The family of 14-3-3 proteins plays an important role in the regulation of cell survival and death. Here, we investigate the role of the 14-3-3 gamma (14-3-3 γ) subunit for glial responses in autoimmune demyelination.

**Methods:**

Expression of 14-3-3 γ in glial cell culture was investigated by reverse transcription polymerase chain reaction (RT-PCR) and immunocytochemistry. 14-3-3 γ knockout mice were subjected to murine myelin oligodendrocyte-induced experimental autoimmune encephalomyelitis (MOG-EAE), an animal model mimicking inflammatory features and neurodegenerative aspects of multiple sclerosis (MS).

**Results:**

Expression studies in cell culture confined expression of 14-3-3 γ to both, oligodendrocytes (OL) and astrocytes. RT-PCR analysis revealed an increased expression of 14-3-3 γ mRNA in the spinal cord during the late chronic phase of MOG-EAE. At that stage, EAE was more severe in 14-3-3 γ knockout mice as compared to age- and gender-matched controls. Histopathological analyses on day 56 post immunization (p.i.) revealed significantly enhanced myelin damage as well as OL injury and secondary, an increase in axonal injury and gliosis in 14-3-3 γ −/− mice. At the same time, deficiency in 14-3-3 γ protein did not influence the immune response. Further histological studies revealed an increased susceptibility towards apoptosis in 14-3-3 γ-deficient OL in the inflamed spinal cord.

**Conclusion:**

These data argue for a pivotal role of 14-3-3 γ-mediated signalling pathways for OL protection in neuroinflammation.

**Electronic supplementary material:**

The online version of this article (doi:10.1186/s12974-015-0381-x) contains supplementary material, which is available to authorized users.

## Background

Oligodendrocyte (OL) damage and astrocyte activation are key features in multiple sclerosis (MS) plaques as well as in inflamed lesions of rodents suffering from experimental autoimmune encephalomyelitis (EAE), an animal model which mimics many histopathological features of MS. To a large extent, inflammatory demyelination is paralleled by loss of OL in the lesions [31–33]. To date, several factors were identified which govern OL susceptibility after an inflammatory attack in the CNS. Such factors comprise neurotrophic cytokines like ciliary neurotrophic factor, leukemia inhibitory factor, or other neurotrophic factors [2, 19, 22]. Besides investigations on glial growth factors, recent studies suggest a role of oligodendroglial death or pro/anti-apoptotic pathways in models of demyelination. Among others, candidates include death pathways involving tumor necrosis factor receptors, Fas-FasL interaction, or tumour necrosis factor-related apoptosis-inducing ligand (TRAIL) [6, 7, 28, 29]. Yet, some of these studies reveal divergent roles of some pathways in toxic vs. autoimmune-mediated demyelination or in vitro vs. in vivo approaches. Thus, it is still tempting to investigate the role of further molecules implicated in the degeneration of CNS cells during autoimmune demyelination. Here, the family of 14-3-3 proteins may be of particular interest. In mammals, 14-3-3 proteins comprise seven distinct highly homologous, ubiquitously expressed isoforms (γ, β, ζ, η, σ, and τ), which represent about 1 % of the total soluble brain protein. 14-3-3 subunits are involved in a wide variety of physiological processes, including neuronal development, apoptosis, cell cycle control, and signal transduction [42]. The extraordinary large number of more than 100 binding partners for 14-3-3 proteins suggests a role as major cellular regulator. To date, a plethora of studies describe a role of 14-3-3 proteins in the pathogenesis of chronic as well as acute neurodegenerative disorders, including Creutzfeldt-Jakob disease, Alzheimer’s disease, amyotrophic lateral sclerosis, or stroke [37]. The function of 14-3-3 proteins in these conditions comprises several mechanisms, which may include basic cellular processes like induction, but also the prevention from apoptotic processes depending on the cell type or disease model studied. To assess the role of 14-3-3 proteins in autoimmune demyelination, we employ mice deficient for the 14-3-3 gamma subunit (14-3-3 γ −/− mice). If not challenged, these mice display a normal anatomy and behavior. Upon inoculation with pathogenic prion protein, survival rates in 14-3-3 γ −/− mice are unchanged [43]. However, 14-3-3 γ deficiency may impair the recruitment of activation-induced cytidine deaminase to immunoglobulin H switch regions and is thus involved in class switch DNA recombination, a mechanism that diversifies the biological antibody response [50].

Here, we investigate the role of 14-3-3 γ protein in glia cell culture and in myelin oligodendrocyte glycoprotein (MOG) peptide 35-55-induced EAE, a model of neuroinflammation with neurodegenerative features. Our data identify a pivotal role of 14-3-3 γ deficiency for OL apoptosis in neuroinflammation.

## Materials and methods

### Glial cell culture

Cell culture experiments were performed with primary murine oligodendroglial precursor cells (OPC) or primary murine astrocytes which were prepared according to established protocols with some modifications [13, 25, 41]. OPC were obtained via anti-O4 MACS sorting and cultured on poly-D-lysine-coated coverslips in serum-free neuro basal medium with addition of NeuroBrew-21 (Miltenyi, Bergisch-Gladbach, Germany).

### Animals

14-3-3 γ −/− mice were backcrossed onto a C57BL/6 background for ten times and bred at the in-house animal care facilities at the University of Ulm, Germany. Control C57BL/6 mice for EAE experiments were derived from the same breeding colony. Female mice for animal experimentation were 10–12 weeks old, and body weight was in a range of 20–25 g. Animals were housed in a room with controlled light cycle and were given commercial food pellets and water *ad libitum*. All animal experiments were approved by the respective state authorities for animal experimentation (LANUV, Recklinghausen, Germany).

### Induction and clinical evaluation of EAE

For induction of EAE, mice received a s.c. injection at flanks and tail base of 200 μg MOG 35-55 peptide (Charité, Berlin, Germany) in PBS emulsified in an equal volume of CFA containing *Mycobacterium tuberculosis* H37RA (Difco, Detroit MI, USA) at a final concentration of 2 mg/ml. Two injections of pertussis toxin (List via Quadratech, UK; 200 ng per mouse post immunization (p.i.)) were given at the time point of immunization and 48 h later. Animals were weighed and scored for clinical signs of disease on a daily basis. Disease severity was assessed using a scale ranging from 0 to 10; scores were as follows [22]: 0 = normal; 1 = reduced tone of tail; 2 = limp tail, impaired righting; 3 = absent righting; 4 = gait ataxia; 5 = mild paraparesis of hind limbs; 6 = moderate paraparesis; 7 = severe paraparesis or paraplegia; 8 = tetraparesis; 9 = moribund; and 10 = death.

### Histology

In the chronic phase of the disease (day 56 p.i.), mice were anesthetized with ketanest and rompun and then were transcardially perfused with saline followed by 4 % of paraformaldehyde. The complete spinal cord was carefully removed, and 8–10 axial sections were further processed for routine paraffin embedding. Paraffin sections were subjected to Bielschowsky silver impregnation, Luxol fast blue, or Cresyl Violet staining to assess parameters of axonal injury, demyelination, or neuronal damage, respectively.

### Immunohistochemistry and immunocytochemistry

Immunohistochemistry was performed with 3 μm paraffin sections as described [21]. If necessary, antigen unmasking was achieved by heat pre-treatment of sections for 30 min in 10 mM citric acid buffer (Mac-3, APP) or 1 mM EDTA (CD3) in a steamer. After inhibition of unspecific binding with 10 % bovine serum albumin (BSA), sections were incubated overnight at 4 °C with the appropriate primary antibody in 1 % BSA. Secondary antibodies were used as indicated below. After blocking of endogenous peroxidase with H_2_O_2_, the peroxidase-based ABC detection system (Vector, Schwalbach, Germany) was employed with DAB as the chromogenic substrate. Specificity of staining was confirmed by omitting the primary antibody as a negative control. T cells were labelled by rat anti-CD3 (Serotec; Wiesbaden, Germany; 1:200); macrophages by rat anti-mouse Mac-3 (BD Pharmingen, Heidelberg, Germany 1:200); astrocytes by rabbit anti-mouse glial fibrillary acidic protein (GFAP) (DAKO, Hamburg, Germany 1:1000); and OL with a rabbit anti-NogoA antibody (Santa Cruz, Heidelberg, Germany 1:100) each with appropriate secondary antibodies (1:200, Vector via Linaris, Dossenheim, Germany). Demyelination was assessed with a mouse anti-mouse 2′,3′-cyclic-nucleotide 3′-phosphodiesterase (CNPase) antibody (SMI91, Sternberger Monoclonals via Covance, Freiburg, Germany).

Proliferating OPC were stained by double labelling with rabbit anti-Olig2 (Merck Millipore, Schwalbach, Germany; 1:500) and mouse anti-Ki-67 (BD Pharmingen, Heidelberg, Germany; 1:100) with Alexa 488 or Alexa 647 conjugated secondary antibodies, respectively (Invitrogen, Darmstadt, Germany). Apoptotic cells were labelled with a rabbit antibody directed against activated caspase 3 (Cell Signaling via New England Biolabs, Frankfurt, Germany; 1:150).

Glial cell cultures were stained via immunocytochemistry with a rabbit anti 14-3-3 γ antibody (1:200; kind gift from Prof. Alistair Aitken, Edinburgh, Scotland) and a mouse anti-GFAP (1:1000), a rabbit anti-NogoA (1:100), or a mouse anti-Olig2 antibody (1:200), respectively.

### Proliferation assay and ELISA

For spleen-cell recall and proliferation assays, single cell suspensions of the spleen from MOG 35-55 immunized 14-3-3 γ −/− mice and wild-type C57BL/6 mice were prepared 10 days after immunization of mice [20]. Cells (3 × 10^5^) were seeded in 96 well microtiter plates (Nunc, Wiesbaden, Germany) in 100 μl medium. Antigen concentrations were 1, 20, or 100 μg/ml for MOG and 1.25 μg/ml for ConA. Triplicate cultures were maintained at 37 °C in a humidified atmosphere with 5 % CO_2_ for 48 h. Supernatants were harvested after 2 days of culture. IFN-γ, IL-17, or IL-4 concentrations were determined by sandwich ELISA kits from R&D or BD as described. For proliferation assays, cells were harvested following a 16-h pulse with 0.2 μCi/well ^3^H-dT (tritiated thymidine, Amersham-Buchler, Braunschweig, Germany). Cells were collected on fiberglass filter paper with a 96-well harvester (Amersham/Pharmacia, Freiburg, Germany), and radioactivity was measured with a 96-well Betaplate liquid scintillation counter.

### Reverse transcription polymerase chain reaction (RT-PCR) protocol

Total RNA from OPC, astrocytes (from cell culture as described above) or total spinal cord was purified over RNeasy columns (Qiagen, Hilden, Germany). Reverse transcription was performed with 12 μl of purified RNA with Quanti-Tect Reverse Transcription Kit (Qiagen). RT-PCR for 14-3-3 γ was performed with commercially available primers (Applied Biosystems) and beta-actin as internal control. All PCR reactions were performed on a 7500 Real Time PCR System (Applied Biosystems) in quadruplicates; relative quantification was performed according to Livak and Schmittgen [24].

### Statistical analysis

Quantitative evaluation of histopathological changes was essentially performed as described earlier [16]. Coded sections were counted by blinded observers by means of overlaying a stereological grid onto the sections and counting inflammatory infiltrates per square millimeter white matter [23]. The extent of demyelination was assessed semi-quantitatively (Image J, Open Source) after CNPase staining on an average of four independent levels of spinal cord per mouse. CD3, Mac-3, or Olig2/Ki67 double positive cells as well as NogoA, GFAP, Cresyl Violet, or activated caspase 3 positive profiles were quantified on three representative sections, each one of the cervical, thoracic, and lumbar spinal cord by counting two defined areas with the most intense pathology under a 400-fold magnification. For quantification of axons in demyelinated white matter lesions of spinal cord cross sections, silver impregnated profiles were counted on six visual fields involving the cervical, thoracic, and lumbar spinal cord using a grid of 100 μm diameter [16]. Data are presented as relative axonal densities.

For statistical evaluation of clinical course, data were pooled from different experiments. Analysis was performed using the Mann-Whitney *U* test *t* test as appropriate depending on normal distribution of data (Prism Graph Pad, La Jolla, USA). All data are given as mean values ± SEM. *p* values were considered significant at *p* < 0.05 and highly significant at *p* < 0.01.

## Results

### Expression of 14-3-3 γ in EAE and in glial cells

**Fig. 1:**
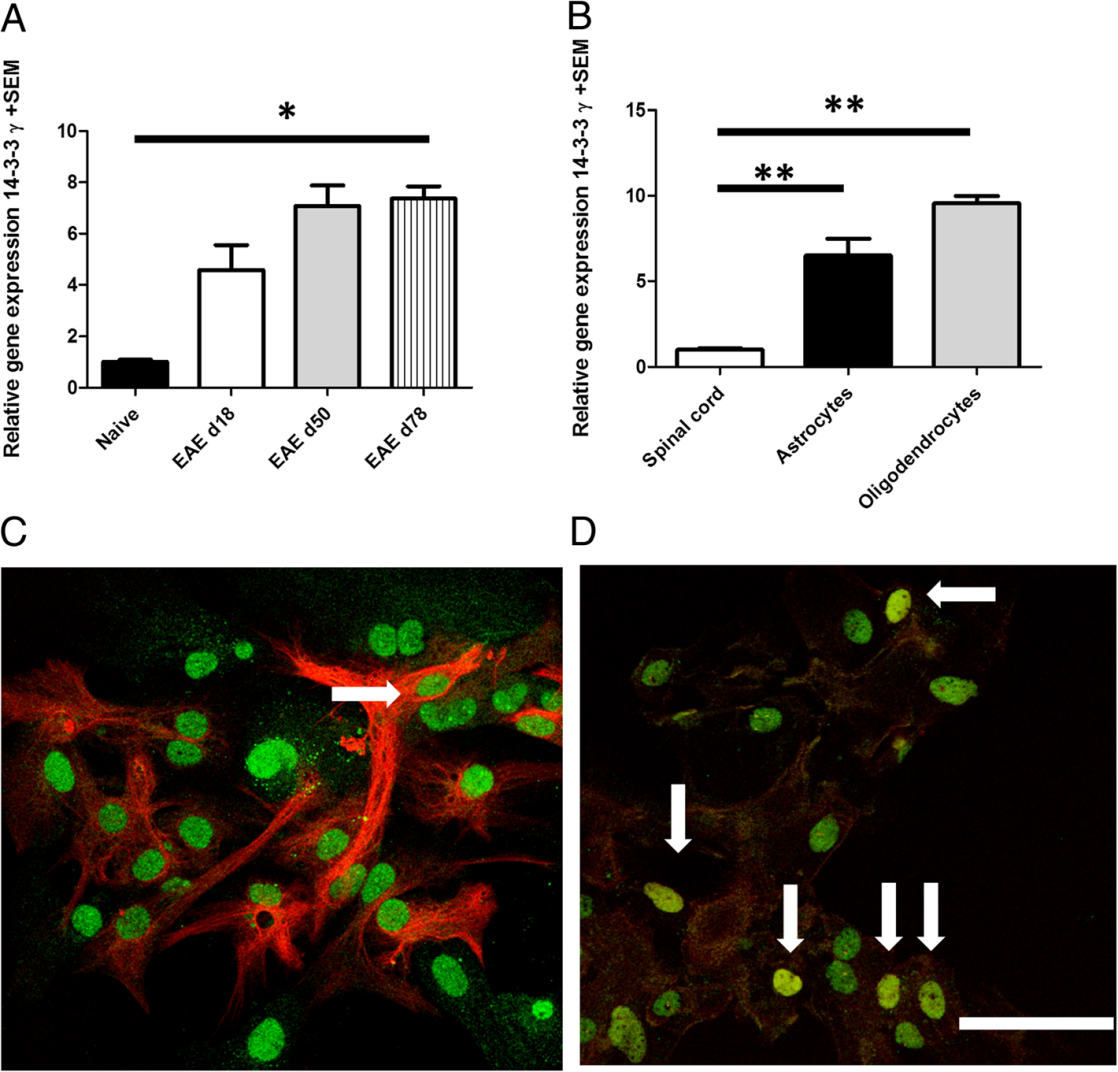
Expression of 14-3-3 γ in glial cells. **a** RT-PCR analyses of spinal cord for the presence of 14-3-3 γ mRNA (*n* = 4 mice per time point). The relative expression in the spinal cord of naïve mice is set to 1. Note the increase of 14-3-3 γ expression in the spinal cord during EAE, particularly at later time points (*p < 0.05 at day 78 of EAE). **b** RT-PCR analysis of 14-3-3 γ mRNA in glial cells as compared to naïve spinal cord (*n* = 4 for spinal cord versus *n* = 9 independent cell preparations for astrocytes and *n* = 3 independent preparations for OPC). Note the significant 14-3-3 γ mRNA expression in glial cells as compared to total spinal cord (**p < 0.01). **c, d** Representative confocal laser scanning images after immunolabeling for 14-3-3 γ with GFAP in primary astrocyte culture **c** or Olig2 in primary OPC culture **d** shows expression of 14-3-3 γ on the protein level in the cytoplasm of some, but not all cells (*arrows* depict double labelled cells, bar = 20 μm)

First, we were interested whether 14-3-3 γ is expressed in EAE. To this end, we compared 14-3-3 γ expression in naïve spinal cord to inflamed spinal cord at different time points of EAE (maximum of disease, day 18 or chronic phase of EAE, day 50 and 78, respectively). RT-PCR analyses of spinal cord revealed an sevenfold increase of 14-3-3 γ expression in the spinal cord during EAE, particularly at later time points (* *p* < 0.05 at day 78 of EAE; Fig. 1a).

Next, we were interested which cells in the spinal cord may have the capacity for 14-3-3 γ expression. Since the increase in 14-3-3 γ expression was most pronounced at later time points of the disease when inflammation has already significantly declined, we focused our analysis on glial cells. RT-PCR for expression of 14-3-3 γ was performed in purified astrocytes and O4 sorted OPC. Expression analyses revealed the presence of 14-3-3 γ in purified OPC as well as in astrocytes with a significant increase as compared to naïve spinal cord (Fig. 1b). To further confine 14-3-3 γ expression to distinct cell types on the protein level, immunocytochemistry after double labelling for 14-3-3 γ and GFAP or Olig2 was performed in glial cell culture. Laser scanning confocal microscopy revealed expression of 14-3-3 γ in the cytoplasm of single astrocytes and OPC while astrocytic processes and some other glial cells were clearly 14-3-3 γ negative (Fig. 1c, d).

### Chronic MOG-EAE in 14-3-3 γ knockout mice is more severe

After having established that 14-3-3 γ protein is regulated in EAE and present in glial cells, we were next interested in its function during demyelination. To this end, we investigated chronic MOG-EAE in 14-3-3 γ knockout mice. Unchallenged, these mice do not display any obvious phenotype and show similar body weights as compared to age-matched controls.

**Fig. 2:**
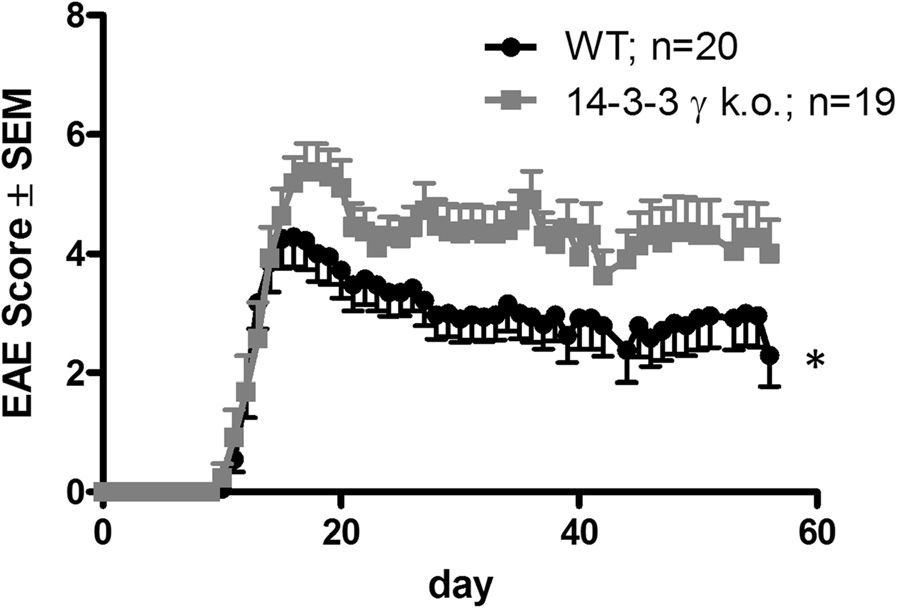
Clinical course of chronic MOG-EAE in 14-3-3 γ knockout mice is more severe. Clinical course of MOG 35-55-induced EAE in 14-3-3 γ-deficient mice (*gray curve*) and C57BL/6 control mice (*black curve*). Data are pooled from three different experiments (*n* = 20 vs. 19 mice per group). Onset of disease is not different between both groups. After the first maximum of EAE in wild-type mice at day 15 p.i.; 14-3-3 γ mice display a more severe course of disease until day 20 p.i.. This enhanced disease severity is maintained in the later disease phase until day 56 p.i., while controls slightly improve over time (*p < 0.05)

After induction of MOG-EAE, disease incidence, mortality, and onset of disease were not different between 14-3-3 γ knockout mice and controls. At the maximum of disease on day 15 p.i., there were no differences between both groups and all mice suffered from severe gait ataxia. Yet, over the following days (16–20 p.i.), 14-3-3 γ knockout mice exhibited an increasing motor impairment with beginning paraparesis while controls already mildly improved. This difference in motor function lasted until the later chronic disease phase (day 56 p.i.) when 14-3-3 γ knockout mice still displayed mild paraparesis as compared to controls which showed mild improvement and only suffered from mild gait ataxia (Fig. *2*).

### Enhanced tissue damage in 14-3-3 γ-deficient mice suffering from chronic EAE

**Fig. 3:**
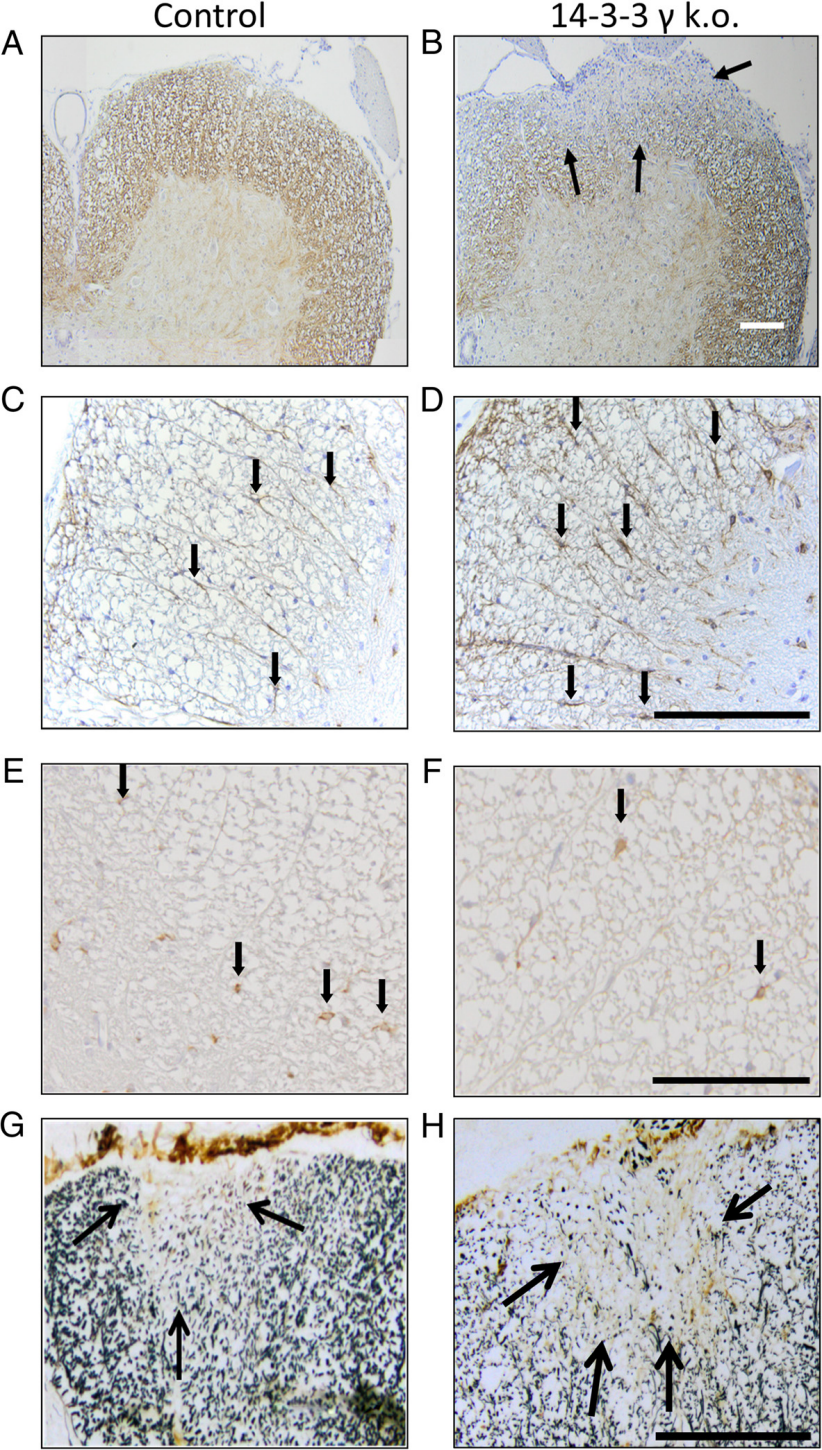
Enhanced tissue damage in 14-3-3 γ-deficient mice suffering from chronic EAE. Representative images of spinal cord cross sections on day 56 p.i. of MOG-EAE are shown. *Bar* represents 200 μm for all sections. Left side (**a**, **c**, **e**, **g**) depicts sections from wild-type, right side (**b**, **d**, **f**, **h**) depicts sections from 14-3-3 γ knockout mice. *Arrows* mark lesions in **b** and **g**, **h**, or labelled profiles in **c–f**. **a**, **b** CNPase staining reveals enhanced demyelination in 14-3-3 γ −/− mice. **c**, **d** GFAP staining reveals enhanced astrocyte activation and gliosis in 14-3-3 γ −/− mice. **e**, **f** NogoA staining reveals reduced numbers of oligodendrocyte around a lesion in 14-3-3 γ −/− mice. **g**, **h** Silver impregnation reveals reduced axonal densities in lesions of 14-3-3 γ −/− mice

To unravel the mechanism behind the more severe EAE course in 14-3-3 γ knockout mice after the first maximum of disease, we performed a histopathological analysis of spinal cord cross sections in the chronic disease phase on day 56 p.i. Upon blinded, semiquantitative analysis of demyelination after Luxol fast blue staining, the extent of demyelination was significantly increased in 14-3-3 γ knockout mice by about 40 %. Similar results were obtained after immunohistochemistry for CNPase as another myelin marker (Table 1; Fig. 3a, b). In a next step, we quantified the numbers of activated astrocytes by immunohistochemistry for GFAP. As compared to controls, 14-3-3 γ knockout mice displayed a 30 % increase in GFAP positive reactive astrocytes in the white matter (Fig. 3c, d and Table 1). At the same time, EAE diseased knockout mice showed a 30 % decrease in numbers of NogoA positive OL in the spinal cord (Fig. 3e, f and Table 1). In naïve 14-3-3 γ knockout mice, the numbers of NogoA positive OL in the spinal cord were not different as compared to age- and gender-matched controls (Additional file 1: Figure S1A–C).

**Table 1:** Blinded quantification of parameters of tissue damage on the spinal cord cross sections after induction of MOG 35-55 (*n* = 6 vs. eight mice per group)

	Control	14-3-3 γ k.o	*p*
GFAP pos. cells/mm^2^ ± SEM	371.8 ± 52.1	595.5 ± 37.0	0.0014
Demyelination of white matter in % ± SEM	3.5 ± 0.55	6.3 ± 0.80	0.037
NeuN pos. cells/mm^2^ ± SEM	290.0 ± 25.2	228.0 ± 16.7	n.s.
NogoA pos. cells/mm^2^ ± SEM	117.2 ± 13.2	72.1 ± 8.5	0.028
Axonal density ± SEM	9.1 ± 0.7	7.1 ± 0.6	0.037

All analyses were done on day 56 p.i. Data are given as mean ± SEM

*n.s.* not significant

*p < 0.05; **p < 0.01

k.o = knockout

Finally, we performed a stereological analysis to quantify axonal densities in demyelinated lesions and densities of alpha motor neurons in the anterior horn. Blinded quantification of axons after silver impregnation revealed a significant 21.5 % decrease of axonal densities in lesions of 14-3-3 γ knockout mice (Fig. 3g, h and Table 1). In contrast, the numbers of neurons in the spinal cord gray matter were not different between knockout mice and controls (Table 1).

In summary, histopathology analysis of chronic EAE reveals enhanced tissue damage in 14-3-3 γ knockout mice with increased demyelination and gliosis, but reduced numbers of OL.

### 14-3-3 γ deficiency does not alter the immune response in MOG-EAE

**Fig. 4:**
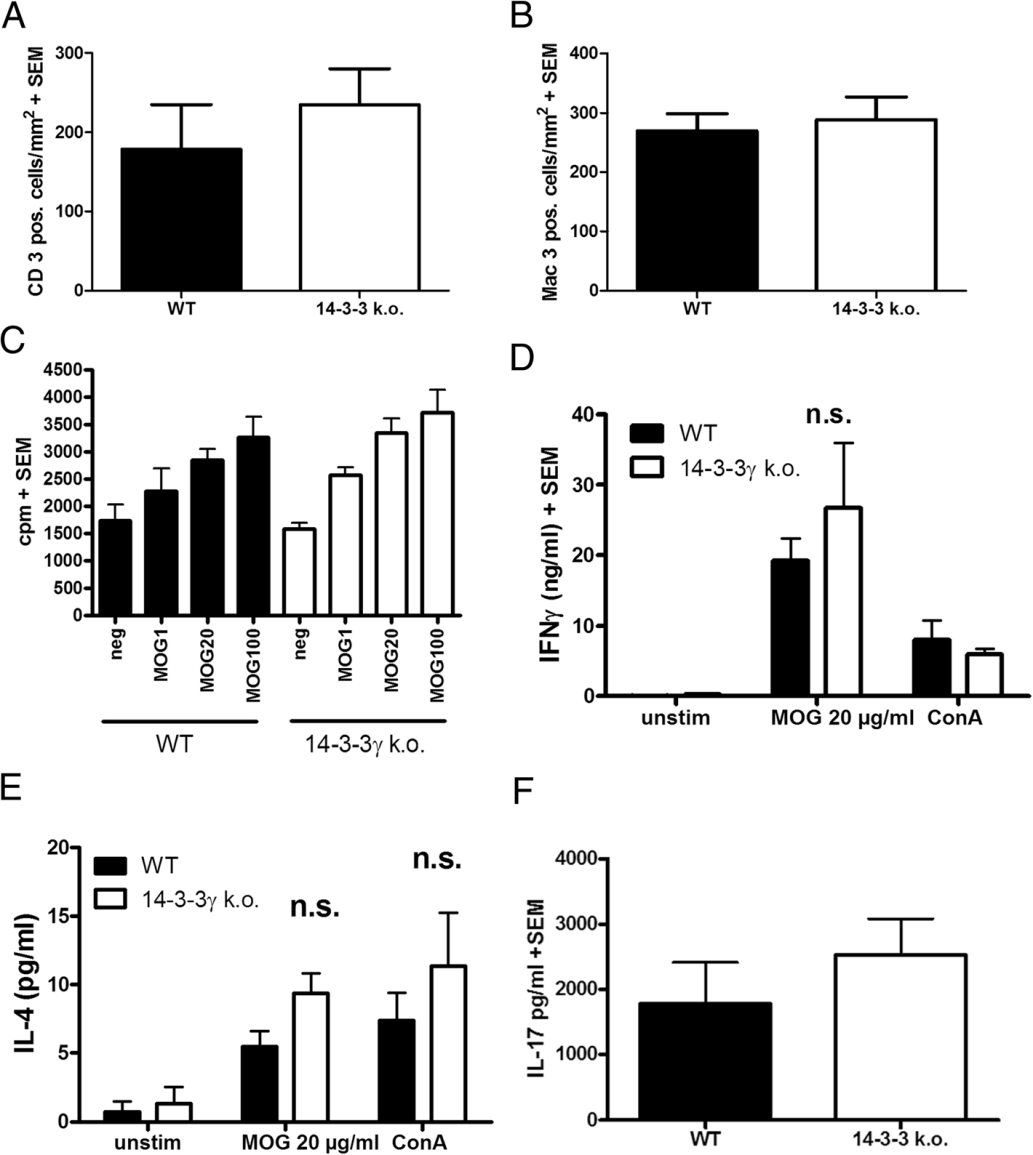
14-3-3 γ deficiency does not alter the immune response in MOG-EAE. 14-3-3 γ knockout mice (*black bars*) are compared to littermate wild-typecontrols (*white bars*). In all assays, there are no differences between both groups. **a**, **b** Blinded quantification of T cells **a** or macrophages/microglia **b** on day 56 p.i after labelling CD3 or Mac-3, respectively (*n* = 6 vs. eight mice per group). **c** T cell proliferation in lymphocyte primary cultures after recall with MOG at different concentrations vs. medium control (neg, *n* = 3 vs. four per group). For MOG, numbers are given as micrograms per milliliter. Proliferation is assessed after labelling with tritiated thymidine, data are given as counts per minute (cpm). **d–f** ELISA-based analysis of cytokine production in supernatants of murine splenocyte primary after recall with MOG antigen or ConA. Analyses comprise quantification of interferon (*IFN*) gamma **d**, interleukin (*IL*)-4 **e**, and IL-17 **f**. *n* = 3 per group. *n.s.* not significant

To investigate the mechanism behind the increase tissue damage in chronic EAE of 14-3-3 γ knockout mice, we performed an immunological analysis. Blinded quantification of CD3 positive T cells and Mac-3 positive macrophages and microglia on spinal cord cross sections at day 56 p.i. showed no differences in immune cell infiltration between 14-3-3 γ knockout mice and controls (Fig. 4a, b). To investigate effects of 14-3-3 γ deficiency on T cell responses in more detail, we investigated T cell proliferation in lymphocyte primary culture and cytokine production in the spleen cell primary culture after immunization with MOG 35-55. In a set of experiments with MOG 35-55 as recall antigen or polyclonal stimulation with ConA, neither T cell proliferation nor production of the Th1 cytokine interferon gamma, the Th17 cytokine interleukin-17, or the Th2 cytokine interleukin-4 was changed in 14-3-3 γ-deficient cultures as compared to matched wild-type controls (Fig. 4c–f).

In summary, the more severe clinical course and enhanced tissue damage in 14-3-3 γ −/− mice after immunization with MOG 35-55 is not governed by an altered T cell response.

### 14-3-3 γ deficiency leads to enhanced OL apoptosis after inflammatory challenge

**Fig. 5:**
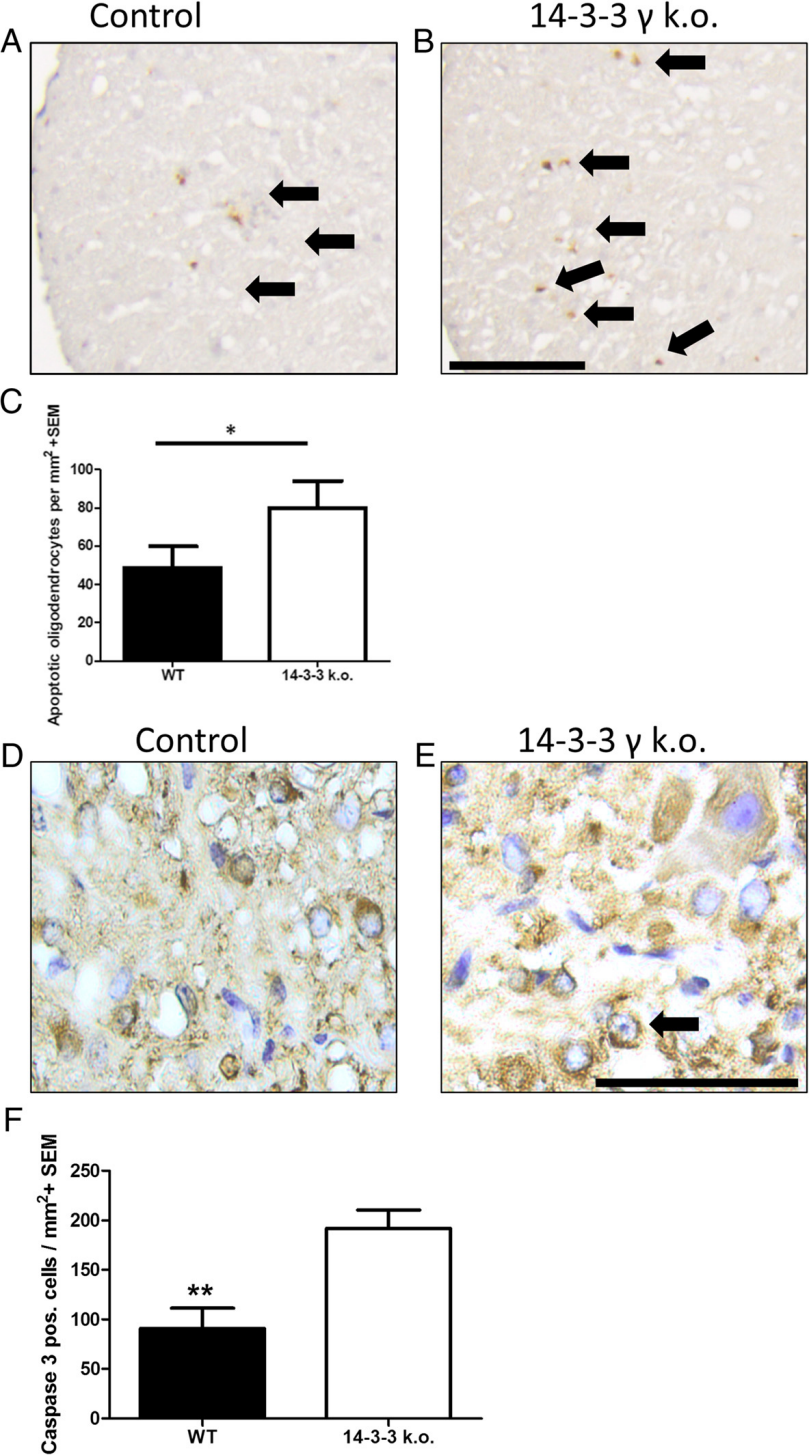
14-3-3 γ deficiency leads to enhanced OL apoptosis after inflammatory challenge. **a**, **b** Representative sections of the spinal cord lesions from MOG-EAE diseased mice (day 56 p.i). Bar 200 μm for all sections. Labelling for activated caspase 3 positive cells reveals an increase in apoptotic cells in 14-3-3 γ knockout mice (see *arrows*). **c** Blinded quantification of activated caspase 3 positive cells on the spinal cord cross sections reveals an increase in apoptotic cells in 14-3-3 γ knockout mice (day 56 p.i., *n* = 6 vs. eight mice per group, **p < 0.01). **d**, **e** Representative sections of spinal cord lesions from MOG-EAE diseased mice (day 56 p.i). Bar represents 50 μm for all sections. Labelling for NogoA and Hemalaun reveals an increase in apoptotic cells in 14-3-3 γ knockout mice (see *arrow* depicting an apoptotic OL). **f** Blinded quantification of apoptotic OL on the spinal cord cross sections after staining for NogoA and Hemalaun reveals an increase in apoptotic OL in 14-3-3 γ knockout mice on day 56 of MOG-EAE (*n* = 6 vs. eight mice per group, *p < 0.05)

The enhanced oligodendroglial and myelin damage in chronic EAE of 14-3-3 γ knockout mice in the setting of an intact immune response points towards glia intrinsic mechanisms responsible for the increased tissue injury. Since 14-3-3 proteins are known to be involved in the regulation of apoptosis, we investigated the effect of 14-3-3 γ deficiency on OL apoptosis after an inflammatory attack. First, we quantified the overall numbers of apoptotic cells on spinal cord cross sections on day 56 of MOG-EAE by staining for activated caspase 3 (Fig. *5*a, b). Upon blinded quantification, 14-3-3 γ-deficient mice displayed a 50 % increase in activated caspase 3 positive cells indicative of apoptosis (Fig. *5*c). To further investigate oligodendroglial cells, we additionally quantified the numbers of OL with pyknotic or fragmented nuclei by morphological criteria after staining for NogoA and Hemalaun (Fig. *5*d, e). Blinded quantification of the spinal cord cross sections on day 56 of MOG-EAE revealed a 40 % increase of apoptotic OL in 14-3-3 γ-deficient mice (Fig. *5*f) indicating that most of the apoptotic cells at this late time of EAE are indeed OL.

In contrast, blinded quantification of Olig2/Ki67 double positive proliferating OPC did not reveal differences between wild-type controls and 14-3-3 knockout mice, neither on day 56 of EAE nor between naïve mice of both genotypes (Additional file 2: Figure S2). In summary, 14-3-3 γ deficiency in autoimmune demyelination is associated with increased apoptosis of oligodendroglial cells but no changes in OPC proliferation.

## Discussion

Our study reveals that 14-3-3 γ protein is present in oligodendroglial cells and that 14-3-3 γ deficiency leads to more severe chronic EAE with enhanced demyelination, OL loss, and increased OL apoptosis. Thus, T cell-mediated neuroinflammation in the setting of 14-3-3 γ deficiency may lead to increased loss of OL which in turn results in increased demyelination, secondary axonal damage, and finally a more severe clinical disease course. These data imply 14-3-3 γ protein as an important factor for OL survival in autoimmune demyelination. Our data on the role of a 14-3-3 subunit in OL extend previous studies on the presence of 14-3-3 proteins in glial cell and particularly astrocytes during development [4] as well as in models of in vitro cell stress, stroke, and also MS lesions [3, 9, 35, 36]. While 14-3-3 subunits and also 14-3-3 γ has been implicated in survival of neurons in different neurodegenerative diseases [37], effects on axonal densities in our model may be rather secondary due to enhanced demyelination and OL damage. Although MOG-EAE may affect neurons in the spinal cord [17, 46], neuronal numbers were not significantly different between 14-3-3 γ knockout mice and controls thus further arguing against a primary effect of 14-3-3 γ on neurons and direct neuronal degeneration in EAE.

Thus, our data add 14-3-3 γ to an array of intracellular signalling molecules or pathways which have already been implicated in OL survival and apoptosis during autoimmune demyelination. Among others, these factors comprise PUMA, BAD, Bcl-2, Akt, p53, TNF receptors, TRAIL, or also IAP family members [9, 12, 14, 15, 28, 34, 39, 40, 49]. Yet, for some of these pathways, divergent data on their role in cell culture vs. in vivo models exist, which points to the importance of testing the relevance of such pathways in different disease models of demyelination. This may be also of relevance for a putative role of 14-3-3 γ for repair or remyelination after a demyelinating attack. Since 14-3-3 γ may protect OL from cell death, it may also facilitate OL repopulation and thus repair either via direct effects on OPC or indirectly via preservation of oligodendroglial cells in or around demyelinating lesions. Yet, repair and remyelination is very incomplete in murine MOG-EAE in C57BL/6 mice [13] and a proper evaluation of these questions need further studies in other, e.g., toxic models of demyelination with better remyelination [18].

The detection of 14-3-3 γ protein not only in OL but also in astrocytes may additionally suggest role of 14-3-3 γ in astrocyte depletion thus indirectly influencing the extent of demyelination as recently discussed in different models after astrocyte ablation [38, 45, 47].

The concept of a protective role of 14-3-3 proteins in oligodendroglial cells may also have consequences for the assessment of 14-3-3 in CSF as biomarker. Here, an increase of 14-3-3 as a bulk maker for neuronal degeneration has been described in several neurodegenerative diseases and also MS [5, 26, 27, 30, 42, 48]. Yet, lacking sufficient reproducibility, the value of 14-3-3 in the CSF as prognostic biomarker in MS remains ambiguous so far [10]. Our data indicate that at least 14-3-3 γ may rather indicate primary damage of oligodendroglia and myelin than injury to neurons or axons in autoimmune demyelination. Thus, further human studies on correlation of CSF 14-3-3 with clinical or magnetic resonance imaging surrogates of myelin damage, e.g., using magnetization transfer ratio or diffusion tensor imaging may be helpful [11].

Finally, our data do not argue for a pivotal role of 14-3-3 γ in immune cells during MOG-EAE, although 14-3-3 adaptor protein-dependent functions have been described in some immune cell-types. In Toll-like receptors pathways of the innate immune response, rather 14-3-3 ε and σ subunits than 14-3-3 γ proteins seem to play a role [1]. In lymphocytes, a role for 14-3-3 proteins has mainly been described in cells of the B cell lineage. Here, the 14-3-3 γ and σ subunits were recently shown to play a role in B cell homeostasis and class switch recombination [44, 50]. However, MOG 35-55 EAE in C57BL/6 mice is a mainly T cell-mediated model of autoimmunity where B cells do not play a vital role for mechanisms of tissue damage [8]. In this study, 14-3-3 γ deficiency did not impact on T cell function including T cell proliferation, cytokine production, and T cell infiltration. Thus, 14-3-3 γ-related immune effects on the neuroinflammation may not be immediately expected based on all available data.

## Conclusion

In summary, we here identify 14-3-3 γ protein as a protective factor for OL in autoimmune demyelination. The characterization of such factors may further improve our understanding on mechanisms of tissue destruction in MS and may eventually contribute to new innovative therapeutic approaches in MS aiming at direct protection of glial cells and myelin or even tissue repair. In this context, future studies in other models of demyelination or with potent small molecule modulators of 14-3-3 pathways are highly warranted.

## Additional files

Additional file 1: Figure S1. 14-3-3 γ deficiency does not influence numbers of NogoA positive OL in naive mice. A, B Representative sections of the spinal cord lesions from naive mice. Bar represents 200 μm for both sections. Labelling for NogoA positive OL does not reveal any difference between 14-3-3 γ knockout mice and controls (see arrows). C Blinded quantification of NogoA positive OL on the spinal cord cross sections reveals no difference between both groups (*n* = 3 mice per group, *p* = n.s.). Additional file 2: Figure S2. 14-3-3 γ deficiency does not influence numbers of proliferating OPC on day 56 of MOG-EAE. Blinded quantification of Olig2/Ki67 positive proliferating OPC on spinal cord cross sections does not reveal any difference between both groups (*n* = 6 vs. eight mice per group, *p* = 0.35).

## Abbreviations

Ci: Curie
CNPase: 2′,3′-cyclic-nucleotide 3′-phosphodiesterase
CNS: central nervous system
ConA: concanavalin A
cpm: counts per minute
EAE: experimental autoimmune encephalomyelitis
IFN: interferon
IL: interleukin
LFB: Luxol fast blue
MOG: myelin oligodendrocyte glycoprotein
MS: multiple sclerosis
OL: oligodendrocyte
OPC: oligodendrocyte precursor cell
p.i.: post immunization
