# Supplementary figures and images for: Role of glial 14-3-3 gamma protein in autoimmune demyelination
Source: J Neuroinflammation. 2015 Oct 6;12:187. doi: 10.1186/s12974-015-0381-x (PMC4595275; doi:10.1186/s12974-015-0381-x)

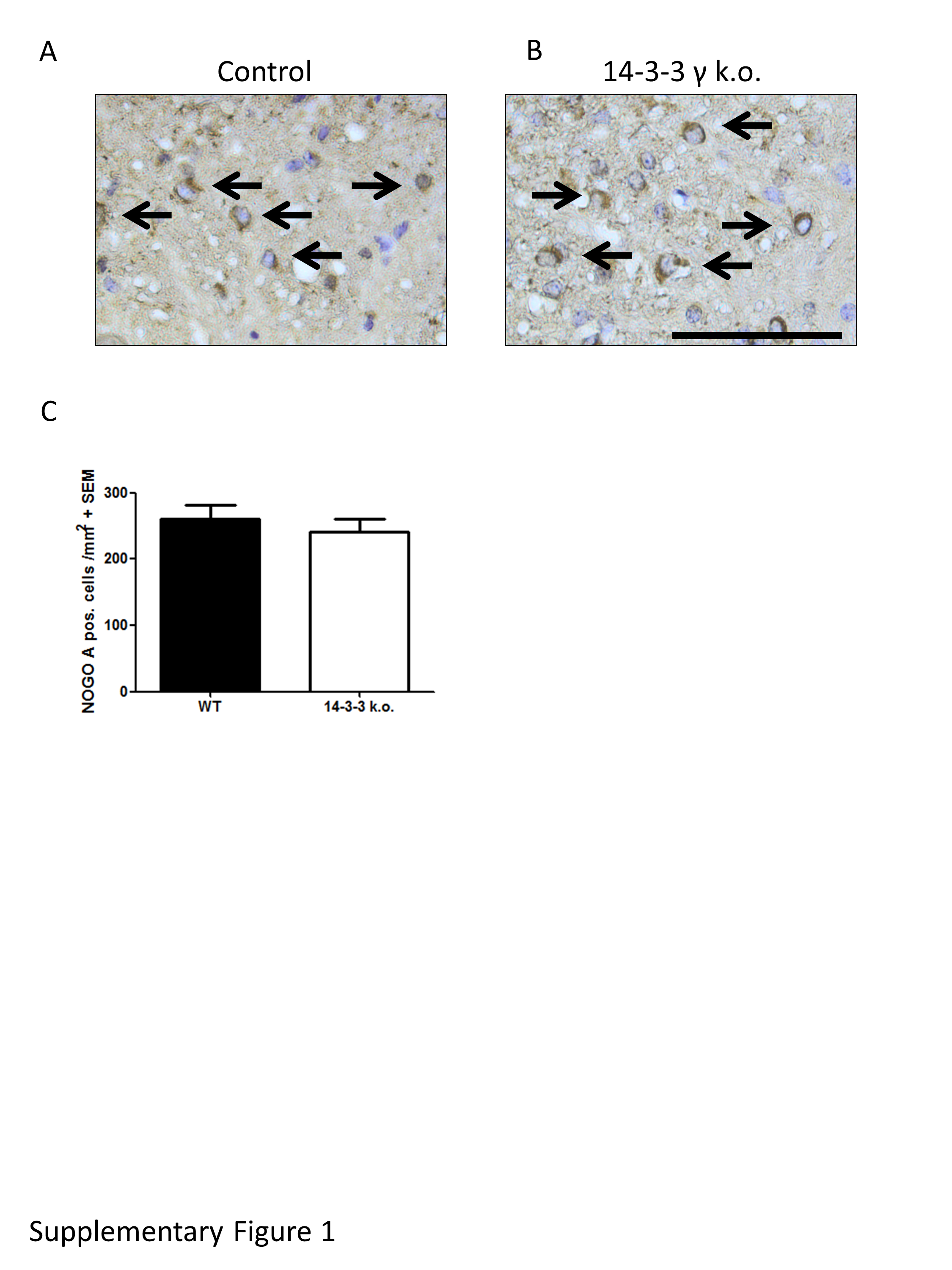

Supplement: Additional file 1: Figure S1. — 14-3-3 γ deficiency does not influence numbers of NogoA positive OL in naive mice. A, B Representative sections of the spinal cord lesions from naive mice. Bar represents 200 μm for both sections. Labelling for NogoA positive OL does not reveal any difference between 14-3-3 γ knockout mice and controls (see arrows). C Blinded quantification of NogoA positive OL on the spinal cord cross sections reveals no difference between both groups (n = 3 mice per group, p = n.s.). [file 12974_2015_381_MOESM1_ESM.tif]

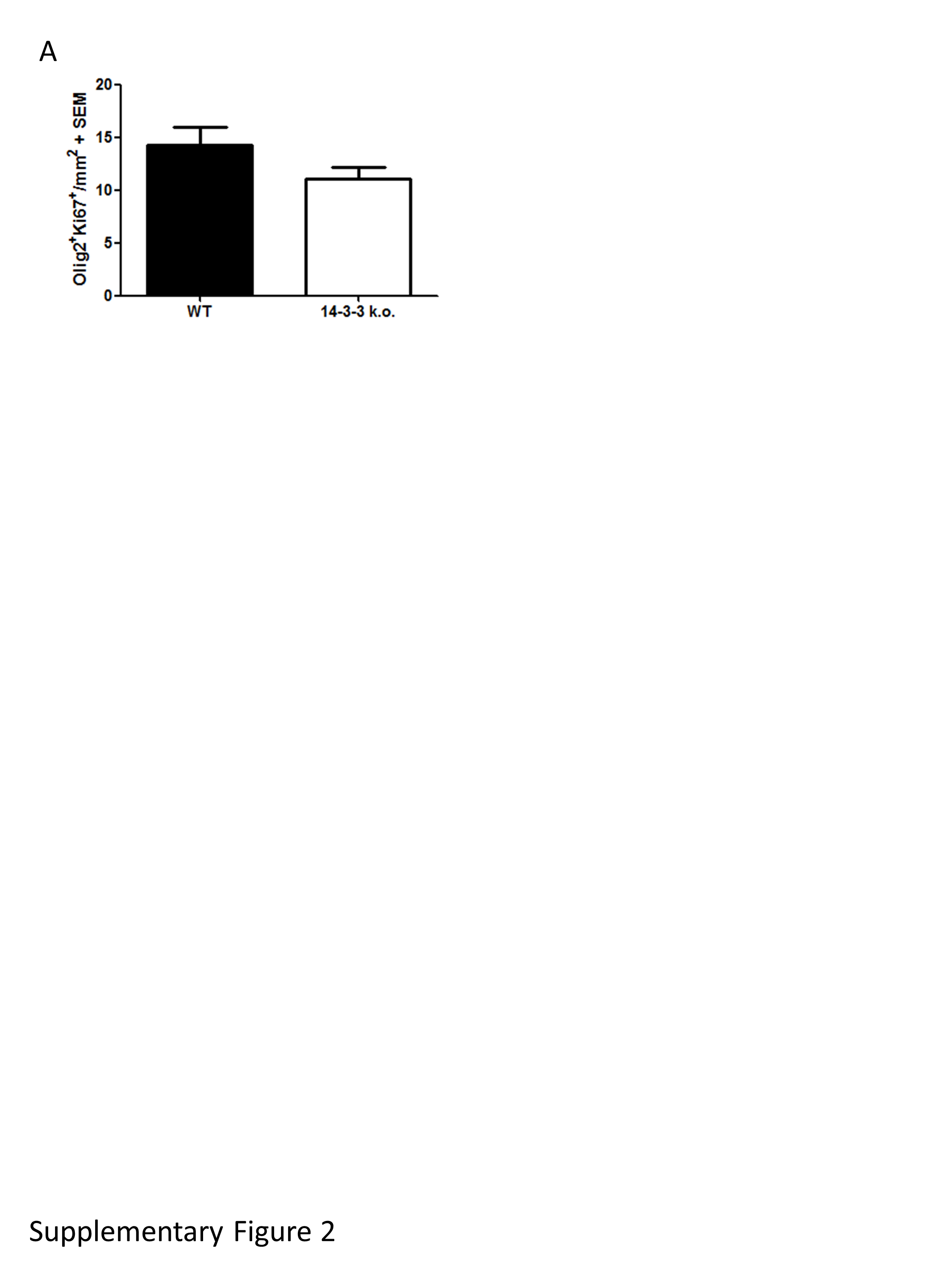

Supplement: Additional file 2: Figure S2. — 14-3-3 γ deficiency does not influence numbers of proliferating OPC on day 56 of MOG-EAE. Blinded quantification of Olig2/Ki67 positive proliferating OPC on spinal cord cross sections does not reveal any difference between both groups (n = 6 vs. eight mice per group, p = 0.35). [file 12974_2015_381_MOESM2_ESM.tif]
